# Determinants of the utilization of youth-friendly sexual and reproductive health services in public secondary schools of Kogi State, Nigeria: an explorative study

**DOI:** 10.1186/s12889-023-15926-y

**Published:** 2023-06-06

**Authors:** Agatha Alami Adione, Nnaemeka Chukwudum Abamara, Bives Mutume Nzanzu Vivalya

**Affiliations:** 1 Department of Public Health, School of Allied Health Sciences, Kampala International University, Western Campus, Ishaka, Uganda; 2 University Health Services, Prince Abubakar University, Ayingba, Kogi State Nigeria; 3 Department of Mental Health and Psychiatry, Faculty of Clinical Medicine and Dentistry, Kampala International University, Western Campus, Ishaka, Uganda; 4 Department of Psychology, Nnamdi Azikiwe University, Awka, Nigeria; 5 Department of Internal Medicine, Masereka General Referral Hospital, North-Kivu, Democratic Republic of the Congo

**Keywords:** Public secondary schools, Youth-Friendly, Sexual and Reproductive Health Services, Utilization, Youth

## Abstract

**Background:**

This study explored the factors associated with the utilization of Youth-Friendly Sexual Reproductive Health (YFSRH) services among school-going Nigerian adolescents.

**Methods:**

This cross-sectional study employed a mixed method involving school-going students attending five public secondary schools in Kogi State, Nigeria. Descriptive statistics were used to determine the patterns of utilisation of YFSRH services, whereas inferential statistics were performed to determine factors associated with utilization of YFSRH services. Qualitative data were analysed by thematic analyses of records using an inductive analysis.

**Results:**

One in two secondary school-going students had used the YFSRH services. Most of the participants had poor awareness of YFSRH services and limited access to YFSRH services. While gender positively predicted the utilisation of YFSRH services among secondary school-going students (aOR = 5.7; 95% CI: 2.4–8.95, p = 0.001), we found that age (aOR = 0.94; 95% CI: 0.67–0.99, p = < 0.001), and religious beliefs (aOR = 0.84; 95% CI: 0.77–0.93, p = 0.001) showed a negative relationship with the utilization of YFSRH services.

**Conclusions:**

Our findings highlight the influence of gender, age, and religion on utilizing YFSRH services. This study recommends the inclusion of sexuality education into secondary school-going student’s curricula, in order to create awareness about the benefit of utilization of sexual and reproductive health services, and this is to encourage young people to utilize the YFSRH services.

## Background

Youth and adolescent sexual and reproductive health (YFSRH) has gained significant insight in the last decades, characterized by increased adolescent sexual activity [1]. The YFSRH services comprise a general understanding of young’s sexual and reproductive lives, interpreted in several levels of the health system. At the individual level, the YFSRH services include a range of activities targeting the special needs of youth, their vulnerabilities, and their desires; associated with high-risk behaviors such as unprotected sexual intercourse and exposure to sexually transmitted diseases [2, 3]. The barriers to accessing sexual and reproductive health (SRH) services utilization in adolescents and young persons included cultural bans, religious influence, financial constraint, lack of adequate information towards the use of the YFSRH services, and negative attitudes of health workers regarding the provision of SRH services to adolescents and young adults [4]. In addition, poor access to YFSRH services in adolescents and young adults is affected by low self-esteem, epistemological gaps, and an unconducive social environment [5]. Although 58.1% of adolescents reported having appropriate information about SRH [6], only 28.1% have access to SRH services utilization [4].

The impaired utilization of SRH services by adolescents and young persons is associated with premature deaths among adolescents [7], which are commonly due to early pregnancy and childbirth. Recent reports showed that nearly 14 million adolescents give birth globally yearly, and more than 90% of these live births occur in developing countries [8], with approximately 70,000 female teenage deaths every year. In these countries, poor access to SRH services among adolescents and young persons is due to a lack of orientation and appropriate information. In Sub-Africa settings, for instance, the majority of young adults lack appropriate information on the utilization of family planning services [9].

Factors affecting the utilization of SRH services among adolescents and young adults vary across studies and include early involvement in unprotected sexual activities with various partners [10], cultural-related limitations [11, 12], and considering sexuality matters as a taboo for adolescents [13]. In several countries, the implementation of sexuality education in schools and universities is affected by religious and conservative political interest groups [14–16]. Consequently, early marriages are still being promoted among adolescent girls living in specific regions of developing countries, such as northern Nigeria, where other research reported one of the highest rates of adolescent’s involvement in early marriage [17]. 43% of Nigerian girls are married before their 18th birthday, and 17% are married before they turn 15 years [18, 19]. Child marriage has high rates (65%) in the North West region and low rates (10%) in the South East [20]. Married adolescents in Nigeria are commonly subjects to blame and discrimination while they look for YASRH services [21]. There is a paucity of data on the factors influencing the utilization of YFSRH services among secondary school-going Nigerian adolescents, where studies have shown that access to needed services is vital in helping prevent adverse SRH outcomes, given that youth and adolescents are less likely to use reproductive health services [20]. Therefore, this study aimed at exploring the factors influencing the utilization of YFSRH services among secondary school-going Nigerian adolescents.

## Methods

### Study design and participants

This cross-sectional study employed a mixed method involving school-going students attending five public secondary schools in Kogi State, Nigeria. These schools were selected as study sites based on their location in a region characterized by high prevalence rates of early pregnancies among adolescents and young adults [22, 23].

**Table 1:** Sample Frame

Selected Schools	Population	Percentage calculated (10%)	Male (60%)	Female (40%)	Total sample
Government Science sec. School Lokoja	1586	158	95	63	158
Gov. Sec. School Abugi	998	79	47	32	79
Gov. Day Sec. School Adankolo	1836	183	108	75	183
Local Gov. Sec. School Otube Karara	1772	177	106	71	177
Local Gov. Sec. School Budon	1662	166	100	66	166
TOTAL	6192	763	456	307	763

Source: field Survey, 2016

We recruited 763 participants enrolled among 8811 secondary school-going students who had registered in the five targeted public secondary schools in Kogi State, Nigeria. The secondary schools are as follows: the Government Science Secondary School Lokoja, the Government Secondary School Abugi, the Government Day Secondary School Adankolo, the Local Government School Otube Karara and the Local Government Secondary School Budon (Table 1). We included secondary school-going students, aged 10 and 24 years, enrolled at each of the five public secondary schools of Kogi state, providing written informed consent or whose tutor/parent provided assent. Exclusions were secondary school-going students not attaining the class during the period of data collection, and having neurocognitive impairment prior to their enrolment for the data collection.

### Procedures

A random sampling, consistent with selecting every fifth on the student list, was performed to select participants and the senior public secondary schools in the study area with an equal chance of participation in the study. Therefore the schools selected are on purpose for the study, and the scientifically determined formula did not apply. The first author approached the chairpersons of the selected schools via a letter, requesting permission to include its students in this study. This letter specified the date of the introduction to participating in this study, the description of ethical considerations, an explanation of consent, the consenting process, and eligibility criteria. Once the permission was granted, school-going participants were contacted in their respective classes. Participants who expressed willingness to participate were asked to provide written informed consent assent after the research assistants offered details about the study. After giving consent, participants were administered a questionnaire by trained research assistants fluent in English. A consent form was shared to all the student participants from the selected public secondary schools in Kogi State Nigeria. They filled out the forms and declare their willingness to participate in the study by writing and endorsing their signature on the form.

We first started by explicitly identifying schools and public health facilities to get acquainted with the facilities management policies that affect youth sexual and reproductive activities. This was then the contact setting where the acquisition of written permission from the State Ministry of Education provided access to the schools from which the sample population was drawn. Managers of government health facilities were visited to solicit permission to involve the youth-friendly sexual and reproductive health unit staff in the study area. Subsequently, contacts were made with the principals of the selected public secondary schools. The permission obtained from the state ministry of education in Lokoja, Kogi State was presented to them. With the principal’s permission, the participants for the study were identified and relevant information was passed on to them regarding the research plans, their expected involvement, and the aim and purposes of the research. Students who provided significant answers to yes/no questions based on access to SRH services and challenges experienced while looking for SRH services were contacted for in-depth interviews, to understand the challenges affecting their utilization of YFSRH services.

Quantitative data which is number-based, countable, or measurable, and it is interpretation-based, descriptive, and relating to language. In this study, quantitative data were collected using semi-structured questionnaires established from socio-demographic characteristics and questions on SRH such as “Have you ever used the SHR?” Each interview took about 15 to 30 min. Data collection was performed in a private setting to complete the questionnaires within the school premises. The data collection tools were pretested among five participants who were not included in the final analyses of this study; and the data collection tools were granted reliability, accuracy, and validation.

Qualitative data which is the descriptive and conceptual findings was collected through questionnaire, interviews, or observations. In this study, qualitative data were collected using in-depth interviews consistent in asking questions such as “Tell us more about your understanding of the YFSRH services”. For a better understanding of the reasons for the non-utilization of YFSRH services, we performed 5 Focus discussion groups (FDGs) involving 16 participants. In the conducted 5 FDGs, the participants were asked “Would you like to tell us about your experience of using the YFSRH services?” The records were interpreted by two independent researchers to get the themes. Data were collected from January to March 2022.

In-depth interviews, the 60% of the participants understand and responded that youth-friendly sexual risk health services involve avoiding indiscriminate sexual relationships, the use of contraceptives such as condoms, pills to prevent sexually transmitted diseases (STDs) and HIV/AIDs, and unwanted pregnancies. 30% of the participants responded that they did not fully understand what youth-friendly sexual risk health services involve, and 10% of the participants were undecided in their responses about having knowledge of YFSRH services.

In the FDGs, the participants were asked “Would you like to tell us about your experience of using youth-friendly sexual risk health services, and 50% of the participants responded that YFSRH service has accorded the opportunity of understanding and knowing what health education is all about within their various environments. 30% of the participants responded that they are hearing about YFSRH services for the first time in the group discussion, while 20% of the participants were undecided in their responses whether they have heard about YFSRH services or not. This is due to the fact that some of them are still very shy to discuss matters relating to sex education.

### Statistical analyses

For quantitative data, descriptive statistics were summarized as proportions or percentages for categorical variables and means/standard deviations or medians/interquartile ranges for continuous variables. Inferential statistics were performed to determine factors associated with utilization of YFSRH services. These factors were included in the regression models based on their potential confounding or influencing effects reported in previous studies [24]. The threshold of statistical significance was set at 0.05. Statistical analyses were performed using SPSS. Qualitative data were analysed using thematic analyses of records using an inductive analysis via Nvivo.

## Results

### Sample characteristics

In the thematic analysis for qualitative data, four themes were recorded from the studies: poor awareness of YFSRH services, cultural bans, and impaired access to YFSRH services including family planning and family permissions. Furthermore, the majority of secondary school-going students had unprotected sexual intercourse. For a 16-year female in SSS1 responded, while asking about the awareness of YFSRH services, reported: “I never been told about these YFSRH services”, while her classmate (17-year male) said “Using the condom impairs the sweetness of the sexual intercourse”. The majority of the participants who were aware of the YFSRH services said “our family members told that we cannot use the YFSRH services such as the use of condoms, the early use of this disrupts the production of children”. A 19-year male in SSS3, said “I usually request for permission from my elder brother before I look for the condom in the nearby pharmacies”.

The majority of school-going students were males; among whom more than half participants reported having visited the family planning services more than once. Most of participants with poor access to YFSRH services were aged between 16 and 20 (34.2%), where high access to YFSRH services was reported in Christian respondents (36.9%) (Table 2).

**Table 2:** Sample characteristics

	Have you ever accessed any youth friendly sexual and reproductive health services?				
	Respondents	No	Yes		
	763 (100%)	N = 381 (49.9%)	N = 382 (50.1%)	Once N = 137 (17.9%)	More than once N = 143 (19.0%)
Gender					
Female	307 (40.0%)	131 (17.1%)	176 (23.0%)	50 (6.5%)	26 (3.4%)
Male	456 (60.0%)	250 (32.8%)	206 (26.9%)	87 (11.4%)	119 (15.6%)
Age in years					
10–15	307 (40.2%)	119 (15.6%)	188 (24.6%)	101 (13.2%)	87 (11.4%)
16–20	261 (34.2%)	140 (18.4%)	121 (15.8%)	100 (13.1%)	21 (2.7%)
21 and over	196 (25.6%)	123 (16.1%)	73 (9.5%)	10 (1.3%)	171 (22.4%)
Religion					
Christian	287 (19.8%)	144 (19.8%)	1433 (19.7%)	120 (15.7%)	23 (3.0%)
Muslim	424 (59.8%)	200 (26.3%)	224 (28.2%)	84 (11.0%)	140 (18.3%)
ATR	52 (9.8%)	38 (4.0%)	14 (2.0%)	4 (0.5%)	10 (1.3%)
Year of student					
SSS1	293 (38.4%)	221 (29.0%)	72 (9.4%)	50 (6.5%)	22 (2.8%)
SSS2	340 (44.6%)	130 (17.0%)	210 (27.6%)	200 (26.2%)	10 (1.3%)
SSS3	130 (17.0%)	30 (3.9%)	100 (13.1%)	50 (6.5%)	50 (6.5%)
Ethnic group					
Igala	162 (21.1%)	82 (10.8%)	80 (10.4%)	50 (6.5%)	30 (3.9%)
Yoruba	157 (20.6%)	67 (8.8%)	90 (11.8%)	20 (2.6%)	70 (9.1%)
Egbira	156 (20.4%)	75 (9.8%)	81 (10.6%)	10 (1.3%)	71 (9.3%)
Bassa	130 (17.0%)	100 (13.1%)	30 (4.0%)	0 (0%)	30 (3.9%)
Other	158 (20.7%)	58 (7.6%)	100 (13.1%)	30 (3.9%)	70 (9.1%)

### Determinant of utilisation of youth-friendly SHR services among secondary going students

At Multivariate analysis, we found that the factors associated with the utilization of YFSRH services among secondary-going students included gender, age, and religion. While gender positively predicted the utilisation of YFSRH services (aOR = 5.7; 95% CI: 2.4–8.95, p = 0.001), we found that (aOR = 0.94; 95% CI: 0.67–0.99, p = < 0.001), and religious belief (aOR = 0.84; 95% CI: 0.77–0.93, p = 0.001) showed a negative relationship with the utilization of YFSRH services (Table 3).

**Table 3:** Multivariate regression analyses of factors associated with the utilization of youth-friendly sexual and reproductive health services

Variables	aOR	95% CI	p-value
Gender	5.7	2.4–8.95	0.001
Age	0.94	0.67–0.99	< 0.001
Religion	0.84	0.77–0.93	0.001
Class of study	1.14	0.67–2.47	0.001
Ethnic group	2.54	0.99–11.40	0.001

## Discussion

This study aimed at exploring the factors influencing the utilization of youth-friendly SRH services among secondary school-going Nigerian adolescents. We found that secondary school-going students had poor awareness of YFSRH services, though they had impaired access to these YFSRH services. This finding supports previous studies indicating limited access to YFSRH services in minority ethnic groups such as Igala, Yoruba, Ebira, and Bassa [24, 25].

Gender disparities are common in many societies and are characterized by a formal demarcation between men and women regarding access to the YFSRH services and increasing the SRH problems and inhibiting access to services [26, 27]. Being male is highly associated with early involvement in recurrent sexual activities, and exposing them to HIV and STDs in adolescents and young adults [28]. Unfortunately, women who are financially, materially, or socially dependent on men lack individual power in negotiating the use of family planning methods during sexual intercourse.

Social expectations about women’s behaviour are underlined in subordinate roles and increase their risk of being sexually assaulted, contracting STDs, and having unwanted pregnancies. Women have also limited access to SRH services compared to men, especially in a vulnerable society [29]. In the African setting, the virginity of a girl until marriage is rewarded and held in high esteem; while the utilization of YFSRH services by unmarried female adolescents is subject to disgrace and is labelled as a social deviant [30–32].

Our results showed that religious belief has a protective effect on the utilization of YFSRH services. This finding is in line with other studies indicating that religion is a significant barrier to adolescents’ utilization of SRH [33–34]. Religious values usually prevent the open discussion of sexual matters which tends to reduce adolescents’ access to basic reproductive health information and services [35]. Although some religious groups have taken action to improve the utilisation of SRH services, the utilization rate of YFSRH services is seemingly very low in several African societies. Interestingly, religion plays a significant role in the utilization of YFSRH Services in Nigeria [36], where two dominant religions namely Christianity and Islam are predominant. However, the Catholic Faith has reservations about contraceptives, encouraging abstinence for unmarried individuals, and natural pregnancy prevention methods [37, 38].

To date, the research on the influence of family religiosity on adolescents’ sexual behaviour remains limited. There is a close association between fundamentalist religious beliefs and reduced sexual activities in some studies, while this relationship is scared in other studies [39]. Furthermore, Catholics advocate the use of condoms in order to prevent HIV infection [40]. While some certain Christian denominations promote family planning, others forbid their members from using contraceptives since it is against their religious permissiveness [41]. Consequently, the utilization rate of SRH services is still alarmingly low in regions with a proportion of Catholics.

Cultural norms and values have a negative effect on the use of SRH services in different communities. They are barriers to accessing and utilizing YFSRH services among adolescents [42]. Cultural factors create an unfavorable environment for the discussion of SRH services due to the firmly rooted sense of condemnation of adolescent sexual activity [43]. Studies have shown that in cultures in which social norms do not condone premarital sex, young people who are unmarried and facing sexual problems such as an STD or unplanned pregnancy will probably address the issue on their own. In communities where premarital sexual activity is not condoned, adolescents have been found to have limited access to SHR Services. In these settings, adolescents are still sexually active despite these moral inhibitions and often have unfavorable outcomes [44]. They may ask for help from trusted friends or siblings or go to private clinics and access care from clinics far from their homes. However, if parents support adolescents, family, and other community members they are better equipped to make healthy choices. In Nigeria, like in other developing countries, adolescents face socio-cultural barriers, making it difficult for them to access and utilize RHS. Many parents in Nigeria do not give children information on sexuality because discussing sex with them is regarded as a cultural taboo. Sexuality education has not been formally introduced into many schools in Nigeria [45]. Adolescents do not have adequate information about SRH and are therefore exposed to a barrage of reproductive health problems.

Finally, the estimated coefficient of the class of study and ethnic group is given as 0.319 and − 0.118 respectively; this shows that a unit increase in the course of study and ethnic group on average brings about a 32% increase in utilization and about 12% decrease in the level of utilization of YFSRH services. However, gender and educational level contributed positively to utilizing YFSRH services. An increase in gender and the level of education are the major determinants of the utilization of YFSRH services among public senior secondary school students in Kogi State, Nigeria. The degree of relationship between utilization of YFSRH services and the health outcomes of school-going students supports the correlation between respondent’s demographics factors and the level of utilization of YFSRH services among public senior secondary school students in Kogi State Nigeria [1, 3, 46].

The major limitation is that this study was conducted in the State of Nigeria, limiting the generalization of its findings to the whole of Nigeria which may have different ethnic groups, different cultures, and beliefs. Another limitation of the study is that some student participants of the study were not very free to respond to the matters of sexuality during the in-depth interview and FGDs as a result of their various religious inclinations that forbids them to discuss sexual behaviour before people. Finally, the study was only conducted with the sample population size of students in SSS2 and SSS1 in the secondary schools selected for the study.

## Conclusion

We found that the utilization of YFSRH services was about 50.1%. Our study indicated that secondary school-going students had poor awareness of YFSRH services and impaired access to YFSRH services including family planning. Our findings highlight the influence of gender, age, and religion on the utilization of YFSRH services. The study concluded that certain policy factors significantly influence the rate of utilization of YFSRH services by the study population. This study recommends the inclusion of sexual education into secondary school-going student’s curricula, in order to create awareness about the benefit of utilization of sexual and reproductive health services, and this is to encourage young people to utilize these services. Rigorous involvement of parents/guardians to break cultural barriers that exist about sex-related topics is also encouraged.

## Data Availability

The data used to support the findings of this study are available from the corresponding author upon request.

## Abbreviations

SRH: Sexual and reproductive health
YASRH: Youth and adolescent sexual and reproductive health
